# Exercise activates lysosomal function in the brain through AMPK‐SIRT1‐TFEB pathway

**DOI:** 10.1111/cns.13114

**Published:** 2019-03-12

**Authors:** Jun Huang, Xue Wang, Yi Zhu, Zhe Li, Yu‐Ting Zhu, Jun‐Chao Wu, Zheng‐Hong Qin, Min Xiang, Fang Lin

**Affiliations:** ^1^ Department of Pharmacology, Laboratory of Aging and Nervous Diseases (SZS0703) Soochow University School of Pharmaceutical Science Su Zhou China; ^2^ Suzhou Vocation Health college SuZhou China

**Keywords:** autophagy, exercise, lysosomal function, TFEB

## Abstract

**Aim:**

To study the effects of exercise on lysosomal functions.

**Methods:**

Mouse exercise model was established and wheel running was scheduled as 18 rpm (14:00‐17:00), 5 d/wk, for 8 weeks. Mice were injected EX527 to inhibit SIRT1 activity. The protein level was assayed with Western blot and immunofluorescence histochemistry. The transmission electron microscopic examination was used to show the structure of lysosome and mitochondria.

**Results:**

Exercise promoted the nuclear translocation of TFEB in the cortex which upregulated the transcription of genes associated with autophagy and lysosome. Exercise directly activated autophagy/lysosome system via up‐regulating of AMPK‐SIRT1 signaling. The SIRT1 inhibitor EX527 decreased TFEB regulated gene transcription but had little effect on the nuclear translocation of TFEB. In addition, long‐term exercise showed more significant effects on activation of lysosomes biogenesis compared with the short‐term exercise and trehalose, a classical autophagy activator in the mTOR‐independent pathway.

**Conclusion:**

Running exercise activates lysosomal function in the brain through AMPK‐SIRT1‐TFEB pathway.

## 1 INTRODUCTION

Physical exercise is considered as an effective strategy to keep body and mind healthiness. Exercise can enhance physical performance, maintain the endocrine and metabolism homeostasis, reduce the risk of cognitive declination, and help people “keep good mood,” which is beneficial to chronic diseases, such as diabetes, hypertension, depression and the neurodegenerative diseases. The advantage of exercise on brain healthiness may be associated with enhancement of neural plasticity, increase of the expression of brain‐derived neurotrophic factor (BDNF) and improvement of cognitive ability after exercise.^1^ However, the mechanism that how exercise influences brain health still needs further study.

Autophagy can recycle proteins, clear protein aggregates, and dysfunctional organelles. Autophagy is a physiological phenomenon, which takes part in the development of organisms, responses to cell stress and keeps the cell hemostasis. Recently, some research has confirmed that exercise activates autophagy, which plays an important role in muscle mass establishment.^2^, ^3^, ^4^, ^5^, ^6^, ^7^

Lysosome is the last step of autophagy flux, so the lysosomal function is crucial for completing the whole process of autophagy. Lysosomes are membrane‐wrapped organelles formed by a layer of phospholipid bilayer.^8^ Inside the lysosomal cavity is an acidic environment containing more than sixty hydrolases, including proteases, peptidases, and phosphatases. Lysosome fuses with autophagosome and endosome to degrade the abnormal protein, dysfunctional mitochondria, and other substances. Lysosomes are also involved in many important biological functions in cells. For example, lysosomes fuse with autophagosomes to form autolysosomes to repair the damaged plasma membranes and respond to the cellular nutritional status and so on.^9^, ^10^ Therefore, a normal function of lysosomes is crucial for the health of cells.

The chronic reduction in lysosome function in brain regions, such as deficits of glucocerebrosidase, is associated with the abnormal accumulation of α‐synuclein in sporadic Parkinson's disease.^11^ Additionally, the deficits of the lysosomal function will cause the aggregation of the abnormal mitochondrial. Since the destructive mitochondria could not be recycled by lysosome, the reactive oxygen species would leak and activate the intrinsic cell death pathway, which was implicated in the neurodegenerative diseases and the lysosomal storage disorders.^12^

Although lysosomal function is very crucial to complete the process of autophagy, little work focuses on the relationship between exercise and lysosomal function. In our previous research, we found chloroquine, the alkalizer of lysosomes, caused disorder of muscle fiber and aggregation of dysfunctional mitochondria. At the same time, exercise could help to decrease the dysfunctional mitochondria and ameliorate the damaged muscle fiber caused by chloroquine.^13^ This research gave us a clue that maybe exercises can enhance the lysosomal function. In this manuscript, we checked the biogenesis and function of lysosome in the brain of the mice treated with long‐term running exercise.

## 2 METHODS AND MATERIALS

### 2.1 Antibodies and reagents

The following antibodies were used for immunofluorescence^14^ and Western blot experiments: LAMP1 (Sigma; #SAB3500285), LC3 (Abcam; #ab62721), β‐Actin (Sigma; #A5441), cathepsin D (SANTA CRUZ; # SC‐377299), Cathepsin L (Abcam; #ab6314), GAPDH (Abcam; #ab9484), TFEB (Sigma; # 3110428), Histone H3 (Cell Signaling; #4499), mTOR1 (Cell Signaling; #2971), p‐mTOR1 (Cell Signaling; #5536), P70S6K (Abcam; #ab32529), p‐P70S6K (Cell Signaling; #9206), AMPK (Cell Signaling; #5831), p‐AMPK (Cell Signaling; #2535), ACC (Cell Signaling; #3676), p‐ACC (Cell Signaling; #11818), ULK1 (Cell Signaling; #8054), p‐ULK1 (Cell Signaling; 5869), DAPI (Sigma; #D9564), Fluoromount™ Aqueous Mounting Medium (Sigma; #SLBN4103V), EX527 (Aladdin Industrial Corporation; #E129892), Resveratrol (Aladdin Industrial Corporation; #R107315).

### 2.2 Animals

The mice were obtained from Shanghai SLAC Laboratory Animal Co., Ltd (Certificate: No. SCXK 2012‐0002), and were kept under laboratory conditions (22°C, 50%‐60% relative humidity, air circulation, 12 hours:12 hours light‐dark cycle) and 5 mice per standard plastic cage. All experiments meet the standard of animal ethical approved by China Medical University. The mice with long‐term exercise training were trained with a wheel running mode in the condition of 18 rpm (14:00‐17:00), 5 d/wk, for 8 weeks. The condition of short‐term exercise training was18 rpm for 1 hour.

### 2.3 Western blot analysis

Mice brain tissue was homogenated and sonicated in RIPA lysis buffer. After centrifuged at 12 500 rpm for 10 min at 4°C, the supernatant was kept as the sample to measure the total protein level with the BCA protein test kit.

The sample proteins were separated by SDS‐PAGE, then were transferred to a nitrocellulose membrane and blocked for 1 hour with 5% no‐fat milk. The membranes were incubated overnight at 4°C with the first antibody. After washing, incubated in fluorescence‐labeled secondary antibody for 2 hour at room temperature. The membrane was processed for protein detection using Two‐color Infrared Laser Imager (LI‐COR company).

### 2.4 Immunostaining and Confocal Microscopy

Mice were perfused with PBS and then with 4% paraformaldehyde. Mice brain was taken out and rinsed with 20%‐30% sucrose for gradient dehydration, then were cut into 10‐μm‐thick sections with a cryotome.

The brain sections were permeabilized with 0.4% PBST (0.4%Triton‐100 in PBS) and blocked with 5% goat serum in 0.4% PBST for 2 hour. After that, the brain sections were incubated with primary antibodies (the dilution of both the antibody TFEB and LAMP1 are 1:500) overnight at 4°C and washed in PBST, and then incubated with Alexa‐conjugated antibodies for 2 hour at room temperature. The sections were observed on an LSM510 Meta Laser Scanning Confocal Microscope (CarZeiss) using a 63‐fold oil mirror.

### 2.5 Transmission electron microscopy

The mice prefrontal cortex was cut into 1 × 1 × 3 mm^3^ and fixed in 2.5% glutaraldehyde phosphate buffer, then were dehydrated with ethanol and acetone. After embedded and dried, the tissue was cut into 70 nm‐thick sections and stained with 3% uranyl acetate citrate. The sections were observed with the HT7700 transmission electron microscope (Hitachi High‐Technologies Corporation) in the Analysis Center of Nantong University (China).

### 2.6 RNA extraction and quantitative real‐time RT‐PCR analysis

Total RNA from prefrontal cortex tissue was extracted with Trizol (TaKaRa, #9109) reagent. The PrimeScript RT Master Mix and ddH2O were added to 500 ng RNA (200 ng/μL) for reverse transcription (37°C, 15 minutes; 85°C, 5 seconds). PCR reaction was performed with SYBR Premix Ex Taq (Takara, #RR420). For the quantitative analysis of PCR results, GAPDH was selected as the internal reference gene. According to the threshold of each gene Ct in the plate, the relative expression of the target genes was calculated according to the following formula:

$$\Delta \text{Ct}=\text{Ct}\;\left(\text{target gene}\right)-\text{Ct}\;\left(\text{GAPDH}\right)$$

The primer sequences are as follows:

**Table:** 

Primer Name	Sequence (5′‐3′)
msGAPDH‐R	5′‐CCTGTTGCTGTAGCCGTATTC 3′
msGAPDH‐F	5′‐TGGAGAAACCTGCCAAGTATG 3′
msLAMP1‐F	5′‐GGTCTGTGGAAGAGTGTGTTC 3′
msLAMP1‐R	5′‐GTTTGCCAGAAAGTGTGCCTC 3′
msLAMP2‐F	5′‐TCTGGAGCAACAGGGAACTG 3′
msLAMP2‐R	5′‐CAGCATAAGCCAGTGCCAC 3′
msTFEB‐F	5′‐GATGTTCTGTGACGCTGGCTG 3′
msTFEB‐R	5′‐GGCAGGCACTAAGTCCAAAC 3′
msMOCLN‐F	5′‐GCGCCTATGACACCATCAAG 3′
msMOCLN‐R	5′‐TATCCTGGCACTGCTCGATG 3′
msV‐ATPase‐F	5′‐AAGCTTCTCAGAGTGGTGGG 3′
msV‐ATPase‐R	5′‐CAGGCAAACAAACACGTGAC 3′

### 2.7 NAD^+^/NADH Quantification

NAD^+^/NADH level was assayed with the EnzyChromTM NAD^+^/NADH assay kit (BioAssay, #E2ND‐100). Homogenize 20 mg brain tissue in a 1.5 mL Eppendorf tubes with either 100 µL NAD^+^ extract buffer or NADH extract buffer, use the supernatant for NAD^+^ or NADH assays according to the protocol, then calculate the ratio of NAD^+^/NADH.

### 2.8 SIRT1 activity assay

SIRT1 activity was assayed with the CycLex SIRT1/Sir2 Fluorometric Assay Kit (MBL, #CY‐1151V2). The cortex tissue homogenizers and reaction buffer were added to a 96‐well black plate (Block Plate, Clear Bottom with Lid, Coring, #3603). The fluorescence intensity, which represents the activity of SIRT1, were assayed with a Full‐Wavelength Microplate Reader (the emission wavelength: 440‐460 nm; the excitation wavelength: 340‐360 nm).

### 2.9 Statistical analysis

Data were presented as the mean ± SD and were analyzed using Prism (version 4) software. Statistical significance was considered when *P* < 0.05.

## 3 RESULTS

### 3.1 Short‐term exercise promoted autophagy‐lysosome level in the brain

**Figure 1:**
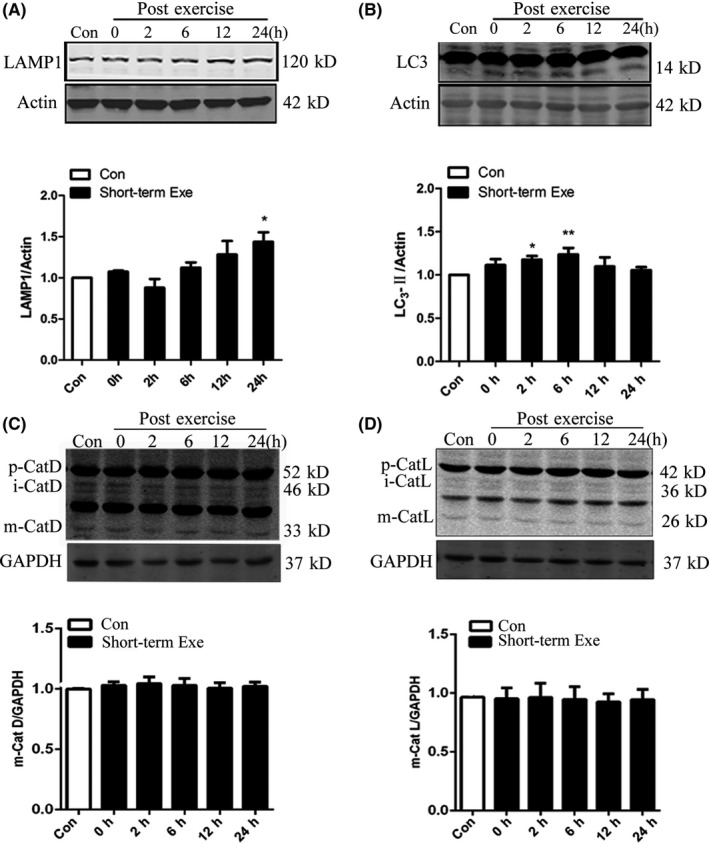
Short‐term exercise promotes autophagy‐lysosome level in the brain. A, B, Western blot of LAMP1 and LC3‐II, Actin was used as the loading control. C, D, Western blot detection of cathepsin D and cathepsin L, GAPDH was used as the loading control. Quantitative analysis was performed with Image J. Values are means ± SD from three independent experiments. *P < 0.05, **P < 0.01, ns *P* > 0.05 vs Control group

To explore the role of exercise on the autophagy‐lysosomal pathway in the brain, we established a short‐term exercise model with mice, and then detected the levels of LC3Ⅱin cerebral cortex of mice. After a short‐term treadmill exercise, we found that the level of LC3Ⅱreached the highest at 6 hours after exercise, and then gradually returned to the normal level (Figure 1A). Similarly, the level of lysosomal membrane protein LAMP1 slightly increased after exercise (Figure 1B). Furthermore, we checked the levels of cathepsin L and cathepsin D. Cathepsin L and D are two important hydrolases in the lysosome, and the levels of their cleaved form can indicate the function of the lysosome. We found that the cleaved forms of both cathepsin D and cathepsin L did not increase after short‐term exercise (Figure 1C‐D). These data suggested that short‐term exercise may activate the formation of autophagosome, but has little effect on the lysosomal function.

### 3.2 Short‐term exercise activated TFEB and enhanced the gene transcription regulated by TFEB in the cerebral cortex

**Figure 2:**
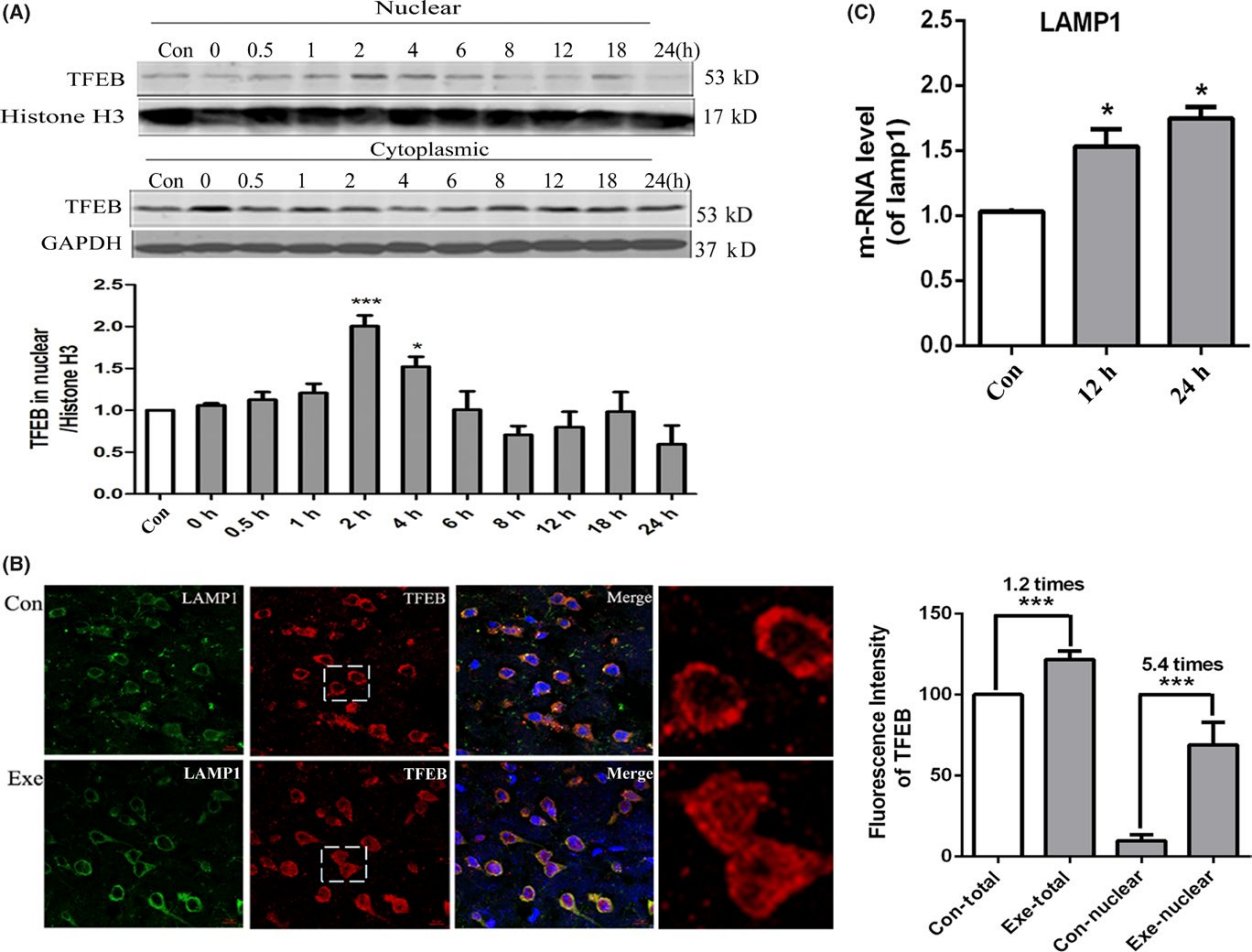
Exercise enhances TFEB nuclear translocation and its gene transcription regulation of in cerebral cortex. A, Western blot analysis of TFEB nuclear translocation at different time points after exercise. GAPDH was used as the cytoplasmic loading control and Histon H3 as the nuclear loading control. B, Quantitative PCR (qPCR) analysis of LAMP1. C, Immunofluorescence images of TFEB in the prefrontal cortex at 4 h after short‐term exercise. Double immunofluorescence of TFEB (green) and LAMP1 (red) was performed. Scale bar = 5 μm. D, Quantitative analysis of TFEB and LAMP1 with Image J. Values are means ± SD from three independent experiments. *P < 0.05, **P < 0.01, ns *P* > 0.05 vs Control group

The transcription factor EB (TFEB) can enter the nucleus and bind to the E‐box of the CLEAR element, which regulates the transcription of the genes associated with the biogenesis of lysosome and autophagosome.^15^ To check whether TFEB in the brain can be activated by short‐term treadmill training, we collected the cortex tissues of mice at different time points after exercise and isolated the nucleus. The Western blot results showed that the TFEB levels in nucleus increased between 2 hours and 4 hours after exercise. In the meanwhile, the TFEB level in cytoplasm decreased after exercise. We also checked the distribution of TFEB in the cerebral cortex at 24 hours after running exercise with the immunofluorescence experiment (Figure 2B). The TFEB level was quantified with the intensity via the Image J software and the results showed that the TFEB level in the nucleus after exercise was 5.4 times than the control. Both the western blot data and the immunofluorescence data suggested that exercise promoted the nuclear transposition of TFEB.

Then, we checked the level of genes whose transcription could be regulated by TFEB with qPCR. LAMP1 is a lysosome membrane protein, and its gene transcription is regulated by TFEB. The data showed that short‐term exercise slightly enhanced LAMP1 mRNA levels (Figure 2C). Together with these results suggested that TFEB nuclear translocation can be induced by short‐term exercise and could potentially be associated with the biogenesis of autophagosome.

### 3.3 Short‐term exercise activates AMPK‐SIRT1 pathway

It is well described that mTOR inhibition activates autophagy. Additionally, it has been reported that the activation of mTORC1 could inhibit the nuclear translocation of TFEB. Thus, we wonder whether the activated autophagy in brain tissue and the nucleus translocation of TFEB induced by short‐term exercise were due to the inhibition of mTOR. We examined the phosphorylation level of mTORC1 and its substrate, P70S6K. Nevertheless, the activity of mTORC1 in mice increased after exercise. This suggested that exercise‐induced TFEB nuclear translocation might be independent of the mTORC1 pathway (Figure S1A,B).

**Figure 3:**
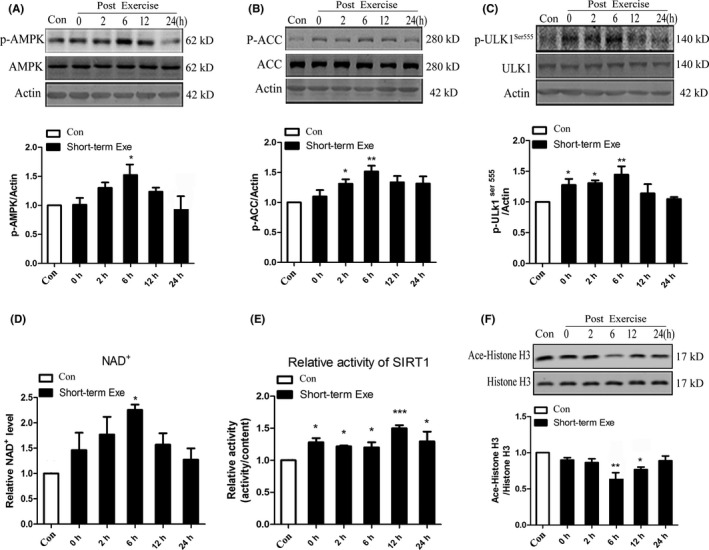
Exercise activates AMPK‐SIRT1 pathway. A, B, Western blot analysis of phosphorylated AMPK and its substrate ACC Ser79. Actin was used as the loading control. C, Western blot analysis of phosphorylation of ULK1 Ser555. Actin was used as the loading control. D, Relative NAD^+^ level of the prefrontal cortex. E, SIRT1 deacetylation activity in nuclear extracts of prefrontal cortex by SIRT1 activity assay kit. F, Western blot detection of the acetylation of Histon H3. Quantitative analysis was performed with Image J. Values are means ± SD from three independent experiments. *P < 0.05, **P < 0.01, ns *P* > 0.05 vs Control group

AMP‐activated protein kinase (AMPK) can be activated directly by exercise in skeletal muscle. To investigate the regulation pathway of autophagy/lysosomal function enhanced by exercise in the brain, we measured the level of AMPK phosphorylation after exercise. The results showed that the level of phosphate‐AMPK increased after exercise and reached the highest level at 6 hours in the brain after exercise, similar to the change of LC3Ⅱ (Figure 3A). To confirm the activation of AMPK, we also measured the level of phosphate Acetyl‐CoA carboxylase(ACC).^16^ ACC is a substrate of AMPK and the level of phosphate‐ACC presents the activity of AMPK. The results showed that the change in phosphate‐ACC after exercise was similar to that of phosphate‐AMPK (Figure 3B), which further confirmed that exercise activated AMPK.

After confirming that exercise can activate the autophagy‐lysosome pathway in the brain, we want to explore the relationship between the activated autophagy and AMPK activation. It has been reported that the phosphorylation of ULK1 at Ser317, Ser555 or Ser777 is essential for AMPK‐dependent autophagy.^17^ We then examined the level of phosphorylated ULK1 at Ser555, and the results showed that the level of p‐ULK1 at Ser555 increased after exercise (Figure 3C). These data suggested that AMPK phosphorylation might participate in the autophagy activation directly by phosphorylating ULK at Ser555 after exercise.

Nicotinamide adenine dinucleotide is a coenzyme, which exists in two forms: the oxidized form abbreviated as NAD^+^ and the reduced form abbreviated as NADH. Phosphate‐AMPK leads to an increase in NAD^+^, and the increase ratio of NAD^+^/NADH can potentially activate the sirtuins. It has also been reported that AMPK regulates energy expenditure by modulating NAD^+^ metabolism and SIRT1 activity.^18^ Thus, we tested the level of NAD^+^ in the cerebral cortex with a kit. The result showed that the level of NAD^+^ in cerebral cortex increased after exercise and lasted to more than 24 hours and the highest point was about 6 hours (Figure 3D). Then, we isolated nucleus of the cerebral cortex and detected the relative activity of SIRT1 level in the nucleus with a kit. The results showed that exercise increased SIRT1 activity in the nucleus (Figure 3E). In addition, we measured the level of acetylation of Histone H3, one of the substrates of SIRT1. The level of acetylated‐Histone H3 decreased after exercise (Figure 3F). All the data were consistent and suggested that exercise activates the AMPK/SIRT1 pathway.

### 3.4 SIRT1 is crucial in TFEB mediated gene transcription after short‐term exercise

It was reported that TFEB can be acetylated at K116 which inhibits the gene transcription, while SIRT1 deacetylates TFEB at K116 and enhances the gene transcription regulated by TFEB.^19^ We wondered whether SIRT1 was associated with the gene transcription induced by TFEB in the brain after exercise.

**Figure 4:**
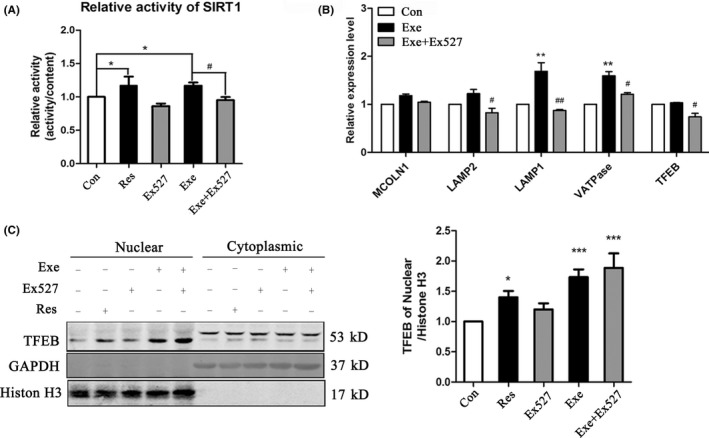
SIRT1 is crucial in TFEB mediated gene transcription after exercise. A, The activity of SIRT1 was measured of prefrontal cortex after the treatment of Resveratrol^5^ or EX527 or Exercise plus EX527. B, Quantitative analysis of gene transcription regulated by TFEB in the prefrontal cortex. Quantitative analysis was performed with Image J. Values are means ± SD from three independent experiments. C, Western blot analysis of TFEB nuclear translocation after treatment with Res or EX527 or Exercise plus EX527. GAPDH was used as the cytoplasm loading control and Histone H3 as the nuclear loading control. *P < 0.05, **P < 0.01, ns *P* > 0.05 vs Control group. #*P* < 0.05, ##*P* < 0.01, ns *P* > 0.05 vs Exercise group

To verify whether SIRT1 is associated with exercise‐induced TFEB nuclear translocation, we tested the effect of SIRT1 inhibitor EX527 and SIRT1 activator resveratrol on the subcellular location of TFEB. We injected resveratrol via the intraperitoneal route and injected EX527 into the lateral ventricle of the mice because EX527 can't go through the blood‐brain‐barrier. Compared with the control, exercise and resveratrol increased the activity of SIRT1, while EX527 inhibited exercise‐induced SIRT1 activity (Figure 4A).

Then, we investigated the level of TFEB in the nucleus with the different treatment. We found that both exercise and resveratrol could promote the nuclear translocation of TFEB. However, EX527 treatment did not prevent the TFEB translocation into the nucleus in the exercising mice group (Figure 4C).

We then tested the transcription level of the target gene regulated by TFEB in the prefrontal cortex. As expected, resveratrol upregulated the target gene levels of TFEB, and exercise could significantly enhance the transcriptional activity of TFEB, too. Among all the target genes, the lysosomal membrane protein LAMP1 and proton pump protein V‐ATPase are the most significantly upregulated after exercise. EX527 inhibited the activity of SIRT1 and abolished the upregulation of LAMP1 and V‐ATPase transcription after exercise (Figure 4B). The results suggested that SIRT1 was crucial in exercise‐induced TFEB mediated gene transcription.

### 3.5 Long‐term exercise activated lysosomes biogenesis

**Figure 5:**
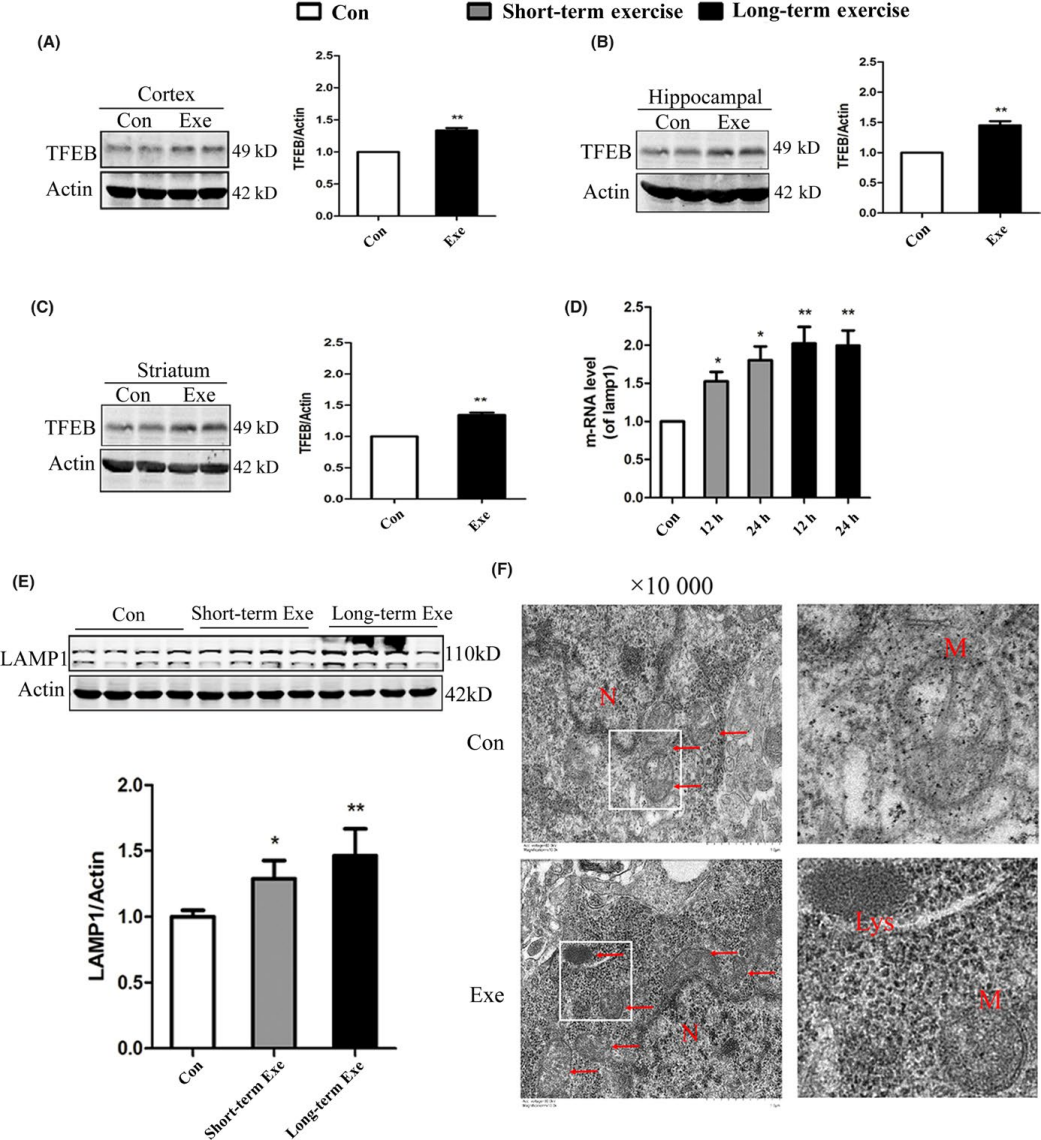
Long‐term exercise is better for activating lysosomes biogenesis than short‐term exercise. A‐C, Western blot detection of TFEB in the prefrontal cortex (A), Hippocampal (B) and Striatum (C). D, Quantitative PCR (qPCR) analysis of TFEB target genes in the prefrontal cortex. E, Western blot analysis of LAMP1 in the prefrontal cortex. F, The electron microscope of the prefrontal cortex (PFC) tissue of mice with long‐term exercise training. AVs, Autophagy vacuoles; Lys, Lysosome; N, Nucleus. Quantitative analysis was performed with Image J. Values are means ± SD from three independent experiments. *P < 0.05, **P < 0.01, ns *P* > 0.05 vs Control group

Although the mature cathepsin D and cathepsin L in cortex after the short‐term exercise had little change, TFEB regulated genes transcription increased. We hypothesized that this discrepancy may be due to the insufficient time duration of exercise. Thus, we wanted to explore whether long‐term exercise had a better effect on lysosomes than short‐term exercise. The long‐term exercise training mice were trained with a wheel running mode in the condition of 18 rpm (14:00‐17:00), 5 d/wk, for 8 weeks. We found that the level of TFEB was significantly increased in the cortex, hippocampus, and striatum of mice after long‐term exercise (Figure 5A‐C). Compared with short‐term exercise, the mRNA level of LAMP1 was significantly elevated to a higher level after long‐term exercise (Figure 5D). Furthermore, we also confirmed that the protein level of LAMP1 after long‐term exercise increased compared with the short‐term exercise group by Western blot analysis (Figure 5E). All those data suggested that long‐term exercise could activate lysosomes biogenesis and enhance the lysosomal function.

In order to monitor the autophagy activation, we used electron microscope to observe the change in autophagosome of the mouse the prefrontal cortex (PFC) tissue after long‐term exercise. In the exercise group, the dysfunctional mitochondria decreased and the number of lysosomes, which have single membrane structure and darker‐colored homogeneous internal, increased obviously (Figure 5G).

### 3.6 Compared with trehalose, long‐term exercise has a better effect in activating autophagy/lysosomal pathway

Trehalose is a well‐accepted activator of autophagy, which acts in an mTOR‐independent mechanism, similar to exercise. So, we wanted to compare the effects of long‐term exercise and trehalose on lysosomal function. We found that the levels of LC3‐Ⅱand LAMP1 in the cortex, hippocampus, and striatum significantly increased in both groups after 8 weeks of the treatment with trehalose or exercise.

**Figure 6:**
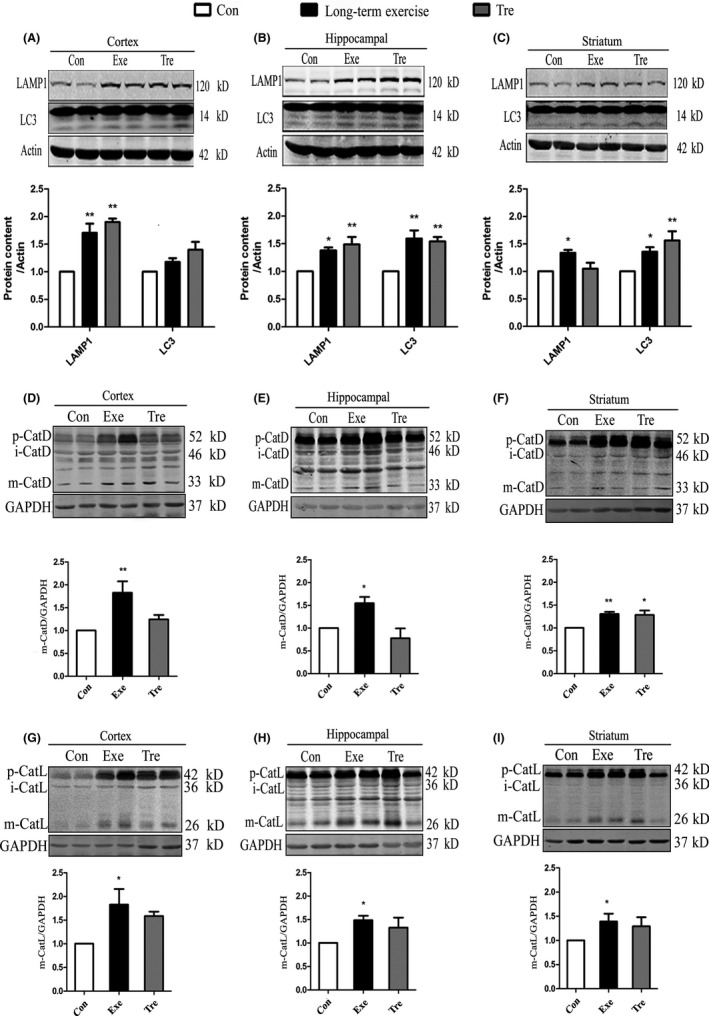
Long‐term exercise is better for activating autophagy/lysosomal pathway than Trehalose. A‐C, Western blot detection of LAMP1, LC3 in Prefrontal cortex (A), Hippocampal (B) or Striatum (C) in the mice with the treatment of exercise or trehalose. Actin was used as the loading Control. D‐F, Western blot detection of cathepsin D in Prefrontal cortex (D), Hippocampal (E) or Striatum (F). GAPDH was used as the loading Control. (G‐I) Western blot detection of cathepsin L in Prefrontal cortex (G), Hippocampal (H) or Striatum (I). GAPDH was used as a loading Control. Quantitative analysis was performed with Image J. Values are means ± SD from three independent experiments. *P < 0.05, **P < 0.01, ns *P* > 0.05 vs Control group

Further, we studied the expression of cathepsin D and cathepsin L in the cortex, hippocampal and striatum of the mice with the two different treatment conditions. As we predicted, compared with the control group, the level of the proenzyme form and the cleaved type of cathepsin D (33 kD) and cathepsin L (26 kD) in the mice brain tissue was significantly increased after long‐term exercise (Figure 6D‐I). Interestingly, after 8 weeks feeding with trehalose, the expression of cathepsin D also increased in the striatum, but not in the cortex and hippocampus (Figure 6D‐I). These data suggested that exercise improved the lysosomal function and that the long‐term exercise has a more significant effect on lysosomal function than trehalose.

## 4 DISCUSSION

Our previous work showed that exercise‐induced autophagy flux could improve the healthiness of skeletal muscle mass.^13^ Lysosomal degradation is a key step in completing the process of autophagy. Therefore, we hypothesized that lysosomal function should be influenced in this process. Here, our results showed that long‐term running exercise could activate autophagy/lysosomal pathway, and enhance the lysosomal biogenesis and function in the brain.

TFEB is the major regulator of autophagy and lysosomal biogenesis.^20^ In our study, we found TFEB in brain translocated into the nucleus and regulated the transcriptional level of genes associated with autophagy/lysosome after running exercise. The EM picture also showed the dysfunctional mitochondria decreased and more lysosome appeared. The results suggested that running exercise could enhance the lysosomal biogenesis in the brain. The lysosomal hydrolase, including cathepsins, plays an important role in the degrading process.^21^ Compared the difference of the cleaved type of cathepsin L and cathepsin D, we proved that long‐term exercise activates the autophagy/lysosomal pathway and the effect is better than trehalose, an autophagy stimulator.

LAMP1 is a membrane protein of lysosome. Therefore, LAMP1 is also a marker of lysosome. In our experiments, we only found LAMP1 had a little increase after long‐term exercise. There could be two reasons. First, LAMP1 not only is a membrane protein of lysosome but also is a substrate degraded by lysosome. The level of LAMP1 increases not only with the enhanced biogenesis of lysosome but also with the dysfunction of the lysosome. For example, chloroquine, which disturbs the lysosomal function by increasing pH, will cause the level of LAMP1 to increase obviously. Second, exercise is a mild method to increase lysosome biogenesis and activate the lysosomal function, which is quite different from results from chemical treatments. Therefore, LAMP1 level can not be a good marker of lysosomal biogenesis by itself, the mature form of cathepsins should also be checked.

Since we have found that exercise increases lysosomal function by improving the nuclear translocation of TFEB and enhancing the transcription of genes regulated by TFEB, then we want to know how exercise enhances the TFEB nuclear translocation. The mTOR1‐dependent pathway is an important mechanism to enhance the function of TFEB.^22^ However, we found exercise activated the biogenesis and function of lysosome in AMPK‐ULK1 pathway but not the mTOR1‐dependent pathway. AMPK maintains the cellular energy homeostasis of various tissues and activates autophagy via phosphorylation of ULK1.^17^, ^23^

Previous studies have found that phosphate‐AMPK can activate SIRT1 and SIRT1 regulates the formation of the autophagic vacuole.^24^, ^25^, ^26^ As so far, we still do not know what occurs after AMPK activation by exercise. However, in glucose deficiency condition, GAPDH is phosphorylated by AMPK and translocated into the nucleus. The phosphorylated GAPDH directly interacts with SIRT1 and activates SIRT1 deacetylase activity.^27^ Some studies also have shown that AMPK and SIRT1 are associated with cell aging and neurological disorders.^28^ It is also be reported that overexpression of SIRT1 has a protective effect on Alzheimer's disease.^19^, ^29^, ^30^ Therefore, we wonder whether exercise could activate SIRT1 in the brain.

The deacetylation effect of SIRT1 on autophagy associated proteins, such as FOXO, Atg1, Atg5, correlates with the increased autophagy level. For example, the acetylated LC3 in the nucleus can be deacetylated by SIRT1 and then redistributed to the cytoplasm and conjugated to autophagic membranes.^31^, ^32^ SIRT1 deacetylated FOXO1, enhancing the gene transcription of Rab7, a crucial important protein associated with the maturation of autophagosomes and endosomes.^33^ SIRT1 also deacetylates FOXO3, leading to the activation of its transcriptional activity and the subsequent Bnip3‐mediated autophagy.^34^ SIRT1‐deacetylated TFEB enhances the expression of genes associated with autophagy/lysosome.^19^ In our experiment, we found the inhibition of deacetylation effect of SIRT1 abolished the transcription of lysosomal genes that regulated by TFEB. The data suggested that exercise activates AMPK‐SIRT1 pathway and the deacetylation effect of SIRT1 is crucial in TFEB mediated gene transcription after exercise.

However, we also found that the deacetylation inhibition of SIRT1 does not inhibit TFEB nuclear translocation induced by exercise, which means there must be other mechanism underlying the enhancement of the nuclear translocation of TFEB. The phosphorylation of TFEB is associated with its distribution in cells. mTORC phosphates TFEB on the lysosomal membrane, and then the phosphorylated TFEB is retained in cytoplasm.^35^ AKT/PKB also phosphorylates TFEB and inhibits its nuclear translocation. The AKT/PKB inhibition activates TFEB and promotes lysosome biogenesis.^36^ In the meanwhile, the phosphatase calcineurin dephosphates TFEB and the dephosphated TFEB would enter the nucleus. Starvation induces the dephosphorylation of TFEB through calcineurin and enhances the nuclear translocation of TFEB.^37^ How does TFEB translocate into nucleus after exercise is not very clear. We will focus on this question in the future study.

Parkinson's disease (PD), Alzheimer's disease (AD), and Huntington's disease (HD) are neurodegenerative diseases featured with the accumulation of specific mutant protein aggregates and protein degradation disorders.^38^ As an “intracellular garbage treatment station,” lysosomal dysfunction is associated with these neurodegenerative diseases.^11^, ^32^, ^39^, ^40^, ^41^, ^42^, ^43^ Upregulation of TFEB regulates the autophagy to improve cell clearance and thus becomes a treatment for lysosomal storage disorders (LSD)^44^ and delays the process of Alzheimer's disease^45^ and other neurological diseases.^16^, ^18^, ^46^

In clinic research, some research suggested that exercise is beneficial to delay the progress of the neurodegenerative diseases.^47^, ^48^ According to our data, exercise can enhance the biogenesis and function of lysosome in the brain, which would be helpful to increase the clearance of the mutant proteins. Long‐term exercise is superior to short‐term exercise or trehalose in promoting autophagy‐lysosomal level. Except for PD, there are not very effective drugs or methods to control these diseases. Therefore, exercise could be a good life style or a “future medicine” to delay the progress of the neurodegenerative diseases and the aggregation of the mutant proteins.

## CONFLICT OF INTEREST

The authors declare no competing financial interests.
