# Supplementary material for: Exercise activates lysosomal function in the brain through AMPK‐SIRT1‐TFEB pathway
Source: CNS Neurosci Ther. 2019 Mar 12;25(6):796–807. doi: 10.1111/cns.13114 (PMC6515701; doi:10.1111/cns.13114)
Supplement: Supplementary file 2 [file CNS-25-796-s002.docx]

Exercise-induced TFEB nuclear translocation is independent on the mTORC1 pathway.
